# Empowering Functional Independence Through Low Vision Rehabilitation in a Patient With Chronic Kidney Disease-Related Optic Neuropathy

**DOI:** 10.7759/cureus.95000

**Published:** 2025-10-20

**Authors:** Naveen K Challa, Abdelaziz M Elmadina

**Affiliations:** 1 Department of Optometry, College of Applied Medical Sciences, Qassim University, Buraydah, SAU

**Keywords:** chronic kidney disease, low vision, optic neuropathy, quality of life, visual impairment

## Abstract

A 32-year-old Saudi male with chronic kidney disease (CKD) on thrice-weekly hemodialysis presented to the low vision clinic with irreversible bilateral optic neuropathy following optic neuritis for low vision services to enhance his daily activities. His distance visual acuity was 20/800 in both eyes, and near vision was J24 binocularly, with no refractive error. Ophthalmological examination showed pale yellow optic discs, preserved color vision, and normal confrontation fields. With low vision rehabilitation, his distance vision improved to 20/80 using a 4x Keplerian binocular telescope, and his near vision improved to J8 with 5x half-eye reading spectacles. He was encouraged to integrate these devices into daily life and referred to ongoing nephrology care. At three months, he reported greater independence in daily tasks and social interactions, feeling more confident in navigation and digital device use. This case highlights how low vision rehabilitation can empower individuals with CKD-related vision impairment, improving their quality of life and functional independence.

## Introduction

Chronic kidney disease (CKD) is a progressive condition characterized by gradual loss of renal function and is associated with significant systemic complications, often requiring long-term dialysis or renal transplantation. It is a growing global health concern with increasing prevalence. Optic neuropathy secondary to CKD is a rare and debilitating condition that significantly impacts visual function and quality of life [1]. Alshehri et al. [2] have shown that the prevalence of CKD in Saudi Arabia is 4.7%, with the most affected age group being 30-39 years. Patients with CKD are at an increased risk of optic nerve damage due to metabolic disturbances, vascular compromise, and associated comorbidities such as hypertension and anemia [3,4]. While the exact mechanism remains unclear, ischemic injury, uremic toxicity, and electrolyte imbalances are thought to contribute to optic nerve dysfunction in these patients [5]. In this report, we present the case of a young adult living with CKD who was referred to a low vision clinic due to significant visual impairment. We aim to highlight how tailored low vision rehabilitation services positively impacted the patient's functional vision, independence, and overall quality of life.

## Case presentation

A 32-year-old Saudi male, a civil engineer who lost his job due to poor vision, was referred to the low vision department of the university medical clinics from the Ophthalmology Department, seeking vision enhancement through low vision devices. The patient reported significant visual difficulties, including challenges in recognizing faces, watching television, and reading distant signs. He also has difficulty in reading printed text and using his mobile phone. At presentation, the patient was diagnosed with irreversible bilateral optic neuropathy secondary to end-stage kidney disease and hypertension and was undergoing hemodialysis three times a week. A renal transplant was also suggested as a treatment option. The patients had an episode of optic neuritis in both eyes a year prior with significant vision loss in both eyes. After treatment by an ophthalmologist patient vision improved from counting fingers close to face to 20/800 in both eyes. However, the vision remained the same after two months of follow-up. A short summary of medical history is presented in Table 1. His systemic history was significant for CKD and hypertension. Over the course of his illness, the patient underwent multiple evaluations and treatments (Table 1). On day 1 of illness, he was diagnosed with stage 5 CKD, optic neuritis, and hypertension, for which he was initiated on thrice-weekly hemodialysis, anticoagulant injections, xanthine oxidase inhibitors, and antihypertensive medications. By day 33, he developed retrobulbar optic neuritis with severe metabolic acidosis; he continued hemodialysis and antihypertensive therapy and was ruled out for multiple sclerosis. On day 77, his condition progressed to end-stage kidney disease, with optic neuropathy attributed to longstanding hypertension and CKD, and he remained on maintenance hemodialysis. By day 120, he was awaiting renal transplantation, with continued dialysis and stable ophthalmic findings, though irreversible optic neuropathy persisted. The latest blood serum report showed increased creatinine and urea with normal electrolytes values. A detailed biochemical profile is presented in Table 2.

**Table 1:** Flow of medical history prior to visit to the Low Vision Department. CKD, chronic kidney disease; HTN, hypertension; MRI, magnetic resonance imaging

Day of Exam	Diagnosis	Treatment
Day 1	CKD, stage 5, optic neuritis, HTN	Hemodialysis three times a week, anticoagulant injections, xanthine oxidase inhibitors, and antihypertensive medications.
Day 33	CKD, retrobulbar optic neuritis, severe metabolic acidosis	Continued hemodialysis three times a week and antihypertensive medications. MRI showed nonspecific bifrontal white matter foci of increased signal intensity. Multiple sclerosis was ruled out (MRI films are not available for review).
Day 77	End-stage kidney disease; optic neuropathy secondary to HTN and CKD	Continued hemodialysis three times a week.
Day 120	End-stage kidney disease; optic neuropathy	Continued hemodialysis three times a week. Suggested replacement for kidney. Waiting for the donor.

**Table 2:** Serum biochemical profile of a CKD patient compared with reference ranges. CKD, chronic kidney disease

Laboratory Parameter	Value	Reference Range	Interpretation
Creatinine	406 µmol/L	60–110 µmol/L	Markedly elevated; consistent with advanced CKD and severe renal impairment
Albumin	50 g/L	35–55 g/L	Within normal range; nutritional/protein status preserved
Urea	18.7 mmol/L	3.6–7.1 mmol/L	Significantly elevated; indicates impaired renal clearance
Sodium	143 mEq/L	136–145 mEq/L	Normal; no dysnatremia
Potassium	4.1 mEq/L	3.5–5.0 mEq/L	Normal; no hyperkalemia
Calcium (total)	2.5 mmol/L	2.1–2.6 mmol/L	Normal; calcium balance maintained though CKD predisposes to disturbances

Multiple sclerosis was ruled out based on magnetic resonance imaging. Following the nephrologist’s recommendation, the patient has remained on hemodialysis since then.

**Figure 1:**
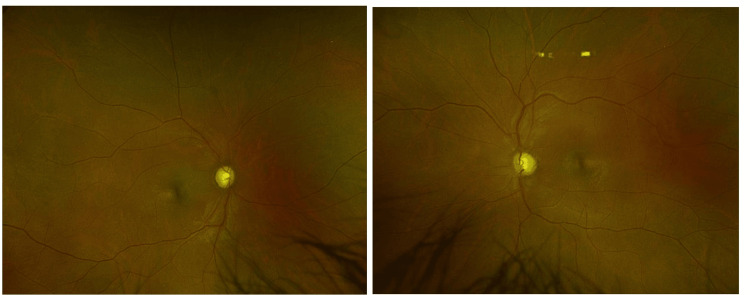
Wide field fundus image of the patient showing the diffuse pale optic disc of both eyes.

At presentation, the patient’s visual acuity was 20/800 in both eyes for distance and J24 at 15 cm with additional lighting. The patient’s objective refraction was OD 0.00/-0.50 x 180° and OS 0.00/-0.50 x 165°, and subjectively, the patient was not accepting any correction. Color vision was normal on Ishihara, and confrontation visual field testing did not reveal any restrictions. Slit lamp examination showed a normal anterior segment in both eyes. Pupillary reactions were sluggish in both eyes, with a relative afferent pupillary defect in the right eye. Intraocular pressure was normal in both eyes. Fundus examination revealed diffuse optic disc pallor in both eyes (Figure 1), with small yellowish drusen-type deposits at the level of the choroid with healthy macula.

A comprehensive low vision evaluation was conducted. For distance, a 4x Keplerian telescope improved visual acuity to 20/80 and was accepted for facial recognition, sign reading, and television viewing. A higher magnification telescope was rejected due to field-of-view constraints. For near tasks, 5x half-eye reading spectacles improved acuity to J8 at 14 cm under enhanced lighting. We tried with other low-vision devices for near, such as a stand magnifier, but the patient was not happy with it and not comfortable maintaining head posture. The patient reported no photophobia or navigational difficulty, and electronic magnification devices were not trialed due to affordability concerns.

The patient received training in handling the telescope for face identification, keeping the print at an appropriate reading distance, using the reading glasses, and using mobile magnification and lighting. The patient was not using any computer. The patient's family was also instructed to improve home lighting and contrast, such as marking stair edges. These strategies were intended to support functional independence and improve safety. He was advised to continue nephrology follow-up for hemodialysis management and to remain engaged with low vision services. At the three-month follow-up, the patient reported significant improvement in performing daily tasks and increased confidence with digital devices. His social interaction and quality of life had improved, showing the real-world value of tailored vision rehabilitation.

## Discussion

The visual impairment in this case was characterized by profound vision loss, sluggish pupillary reflexes, and optic disc pallor, aligning with previous reports of CKD-associated optic neuropathy reported in other countries [2,6].

Low vision rehabilitation plays a crucial role in maximizing functional vision in patients with optic neuropathy. Telescopes and high-powered reading magnifiers can greatly improve visual performance for both distance and near for individuals with severe vision loss. In this patient, the 4x telescope improved distance vision from 20/800 to 20/80, enabling face recognition and reading distant signs, while 5x half-eye reading spectacles enhanced near vision to J8, facilitating reading and computer use. These findings support the evidence that optical magnification remains a viable strategy for enhancing residual vision in optic neuropathy. Half-eye spectacles provide a broader field of view and a more discreet, aesthetically pleasing design compared to other low-vision devices. They were especially beneficial for writing tasks. However, they do come with certain limitations, such as a short working distance and a limited depth of field, and may not be suitable for individuals with postural challenges [7].

The advancement of electronic portable devices has significantly improved near visual acuity in individuals with severe visual impairments [8]. CCTV offers several advantages, including maximum magnification, adjustable illumination, optimal operating distance, and enhanced text contrast. However, CCTV requires some initial training and adaptation, and a notable drawback is its high cost, which may limit accessibility for many patients.

Contrast enhancement and adaptive lighting help people with contrast sensitivity loss due to optic nerve disease, as there is contrast sensitivity loss in these patients. The patient's improved vision with additional lighting in the current case highlights the importance of adjusting the environment to enhance visual performance. Providing guidance on home adaptations, such as improving illumination and adding contrast to stair markings, is an essential part of best practices in low vision management.

## Conclusions

Given the progressive nature of CKD, ongoing multidisciplinary collaboration between ophthalmologists, nephrologists, and low vision specialists is essential. Regular renal monitoring and hemodialysis may help mitigate further visual decline, while continued low vision rehabilitation can enhance daily functioning. Proper awareness and guidance for patients are equally important, as they help individuals cope more effectively with the challenges of combined systemic and visual disability. Appropriate counselling is also greatly needed for all patients, as it provides emotional support, improves coping strategies, and encourages adherence to rehabilitation. This case highlights the impact of vision rehabilitation in enhancing daily function and overall well-being of patients with severe vision loss in CKD.
